# Discrepancy Between Gaze Fixation and Risk Perception in Fall Risk Scenarios Among Healthcare Professionals and Students

**DOI:** 10.7759/cureus.93091

**Published:** 2025-09-24

**Authors:** Katsuhiko Arihisa, Hideki Miyaguchi, Tomoko Ohura, Chinami Ishizuki, Ryohei Kishita, Wataru Matsushita

**Affiliations:** 1 Faculty of Allied Health Sciences, Kansai University of Welfare Sciences, Kashiwara, JPN; 2 Faculty of Health Sciences, University of Kochi Health Sciences, Kochi, JPN; 3 Center for Gerontology and Social Science, National Center for Geriatrics and Gerontology, Obu, JPN; 4 Faculty of Health Sciences, Osaka University of Human Sciences, Suita City, JPN; 5 Department of Service Development, Sunwels Co. Ltd, Fukuoka, JPN

**Keywords:** eye tracking, fixation time, hazardous situation, healthcare workers, medical error, near-miss and adverse event, risk perception

## Abstract

Background

Medical errors and near misses remain critical patient safety concerns. While eye-gaze analysis has been used to study risk perception among healthcare professionals, the relationship between visual fixation time and actual risk judgment under time constraints remains unclear, particularly regarding experience-based differences in hazard recognition. Understanding healthcare workers' risk perception strategies is essential for effective training in preventing medical near-misses and adverse events in healthcare settings. This study addresses two crucial research questions that delve into the characteristics of risk perception when observing hazardous situations. It explores whether gaze fixation time on risks differs with experience and whether the tendency to overlook fall risks (hazards) despite observing them decreases with expertise.

Methods

The study involved 23 healthcare workers from three hospitals with convalescent rehabilitation units and 25 fourth-year students from rehabilitation training schools in Japan. The participants wore eye trackers and completed the Time Pressure-Kiken Yochi Training Effectiveness Measurement System (TP-KYT), which assesses hazard prediction skills. Healthcare professionals were required to have: (i) continuous clinical practice, (ii) TP-KYT scores ≥ 138 points (competency threshold), and (iii) analyzable eye-tracking data. Students were included if they were: (i) fourth-year rehabilitation students, (ii) able to continue academic activities, and (iii) had analyzable eye-tracking data. No TP-KYT score criterion was applied to students. We compared TP-KYT scores with gaze fixation duration, total amplitude of saccades, and fixation and non-cognition. We tested TP-KYT scores, gaze fixation duration, and total saccade amplitude using the Mann-Whitney U test. We examined fixation and non-cognition using risk ratios.

Results

Healthcare workers had higher TP-KYT scores than students (p < 0.01). Students had a shorter total fixation duration on hazards in one item of Scene 4 (p = 0.02), but there was no difference in total amplitude of saccades between groups in any item. Students demonstrated a significantly higher likelihood of not perceiving a risk despite fixating on it (Fixation and non-Cognition, FnC), particularly for environmental and contextual hazards (e.g., Scene 3 risk ratio for FnC: 2.68 (95%CI 1.46-4.91) for students vs. healthcare workers).

Conclusion

A key characteristic of student risk perception is an inability to contextualize relationships between objects and environmental factors. Risk education should therefore focus on teaching recognition methods that incorporate contextual understanding. These findings can inform the development of training strategies aimed at improving healthcare safety by preventing adverse events.

## Introduction

Since the Institute of Medicine published "To Err Is Human: Building a Safer Health System" in 1999 [1], efforts to reduce medical errors and other adverse events and improve patient safety have received much attention. Medical near misses/adverse events occur among rehabilitation, allied healthcare professionals, and medical professionals such as physicians and pharmacists [2]. Heinrich's Law describes accidents as follows: "For every major accident, there are 29 minor accidents, and for every minor accident, there are 300 near misses [3].

The most commonly reported causes of accidents among healthcare professionals are errors in judgment, lack of preparation, lack of experience, lack of knowledge, and lack of interprofessional communication [4]. One factor is the healthcare professionals’ perspective, which includes risk-specific cognitive biases. Risk perception is a decision-making process regarding how to deal with anticipated risks and includes cognitive, affective, contextual, and individual factors [5]. Cognitive factors are related to how individuals perceive and understand the magnitude and likelihood of harm from risk. Affective factors refer to the phenomenon where emotions, such as fear, exert a significant influence on risk judgment. Cognitive and affective factors include individual factors such as cultural characteristics, gender, age, personality traits, past experiences, and contextual factors such as the framing of risk information and the availability of alternative sources of information. These factors complement and mitigate risk perception [5,6]. Identifying the risk perception strategies of healthcare professionals is essential to prevent medical near-misses and adverse events.

One method used to confirm risk perception is an objective study using eye-gaze analysis. Research using eye gaze analysis began with research on the safety of driving [7-9] and is now expanding to health and medical fields, focusing on medical near-miss and adverse events [10-12]. A study on nurses was conducted to explore human risk perception strategies by comparing the gazes of experts and novices. Gaze shifts and duration in gaze analysis are essential in selective visual information acquisition, and the ability to avoid hazards is thought to vary with experience [13]. In a previous study on gaze analysis for hazard prediction in nursing, novice and experienced nurses were presented with still images of a hospital room. Their eye movements were analyzed using an eye-tracking system [14]. The results of a subsequent interview survey showed that although there was no significant difference in gaze duration, experienced nurses reported more specific stories about anticipatory reasoning and positional assessment for hazard prediction than new nurses. Another study compared the gaze time of nursing students and professional nurses in hazardous situations and reported that the gaze time of nursing students was 1.22 times longer than that of experienced nurses. The time to prevent a patient from falling and secure the patient was longer [15].

Furthermore, in a study that examined the hazard perception characteristics of nurses and nursing students within a 10-second time limit, nurses reported that they recognized more hazards and could accurately identify them even within a short period [16]. Although an expert's eye movements are small, environmental information is complicated in medical situations. Therefore, it is necessary to make risk judgments while constantly observing the relationship between the environment and people. This may only sometimes be the case when an expert's eye movements are small.

Thus, when trying to understand risk perception using gaze analysis, it is often compared with the time to risk detection or the time to gaze, and gaze and risk perception have been treated as equivalent [13-16]. However, a longer gaze time does not necessarily mean the risk is accurately perceived, and gaze time may be prolonged by accurately identifying the risk [17]. Therefore, from a medical near-miss and adverse-event prevention perspective, risk perception, a subjective judgment, must be considered. When time pressure is applied to risk perception, influential factors are perceived, a more pronounced difference in risk perception occurs, and a difference in the judgment of experts and non-experts appears [18]. Moreover, it is predicted that less-experienced users will spend more time looking at an object and will be more likely to be unaware of the situation, even if they are looking at it.

Although there is already established knowledge about the differences in the amount of time individuals spend looking at dangerous situations based on experience and the differences in the time it takes to detect a dangerous situation within a time limit [15,16], not much is known about examining the amount of time individuals spend looking at dangerous situations when there is a time limit, or whether or not they accurately judge what they see within the time limit as a dangerous event.

In this study, we will determine whether the time spent looking at the hazard depends on experience and whether experience reduces the risk of missing a fall hazard despite looking at the situation. By conducting this study, we can confirm in detail the information needed to understand students' risk perceptions and provide a theoretical basis for more objective educational interventions.

## Materials and methods

The study was approved by the Ethics Committee of the Fukuoka School of Health Sciences, International University of Health and Welfare, Japan (approval number: 14-Ifh-08). Written and verbal explanations were provided to the study participants, and informed consent was obtained from all participants.

Study population

The participants fell into two groups: healthcare professionals and students. Healthcare professionals were recruited from a total of 244 professionals working in the rehabilitation departments of three recovery-phase rehabilitation hospitals in Japan (Kinkai Rehabilitation Hospital: 65; Kashiigaoka Rehabilitation Hospital: 95; Chidoribashi Hospital: 84). Of this pool, 34 professionals who were available to participate during their work hours and provided consent were enrolled. The inclusion criteria for this group were: (i) holding a national license as a physical therapist, occupational therapist, or nurse, and (ii) having at least one year of full-time clinical experience. Students were recruited from a pool of 92 fourth-year occupational therapy students at one university between 2016 and 2021. From this pool, 29 students who provided consent were enrolled. The inclusion criterion was being enrolled in the final year of their program and having completed all required coursework, as well as at least one clinical clerkship.

For both groups, participants were required to be free of any self-reported physical or mental health conditions that would impair their ability to complete the study tasks. For healthcare professionals, a score of at least 138 on the Time Pressure-Kiken Yochi Training Effectiveness Measurement System (TP-KYT) [19], a tool for assessing hazard prediction skills under time pressure, was required to meet the standard for competence. Therefore, participants scoring below this threshold were excluded, although the said criterion was not applied to students. Participants from both groups were excluded if they failed to meet the criteria for reliable eye-tracking data.

Materials and equipment

Eye Tracking Device

This study used a Tobii Pro Glasses 2 50 Hz wearable wireless eye tracker (Tobii AB, Stockholm, Sweden). The system uses a camera built into glasses to measure eye movements. The camera records corneal reflections from infrared radiation to track the position of the pupil and superimposes the subject's focus of attention on the video recording. Eye trackers cannot be used with eyeglasses; therefore, eyeglass wearers were compensated for their vision by using a set of G3 lenses made exclusively for eye trackers (Tobii AG).

Hazard Recognition in Fall Hazard Situations

The TP-KYT is an assessment method that can quantitatively measure the ability of healthcare workers to anticipate danger and has been confirmed in terms of validity and reliability. This tool illustrates five scenes frequently associated with falls and quantifies the degree to which fall risk can be accurately and rapidly assessed. For each scene, participants were allotted 10 seconds to identify and report hazards for that scene. Scores were assigned based on the number of correctly identified pre-determined hazards, with critical hazards weighted more heavily, while no points were awarded for identifying non-hazardous areas. The total score reflects overall hazard prediction ability [19]. Participants were asked to check the areas they felt were dangerous in a specified scene within 10 seconds and describe why they felt so. The required time for the TP-KYT was measured using an AD-5701A timer (A&D Co. Ltd., Tokyo, Japan), starting the 10-second measurement only after giving the signal to turn the page. 

Area of Interest and Gaze Hold Settings

**Figure 1:**
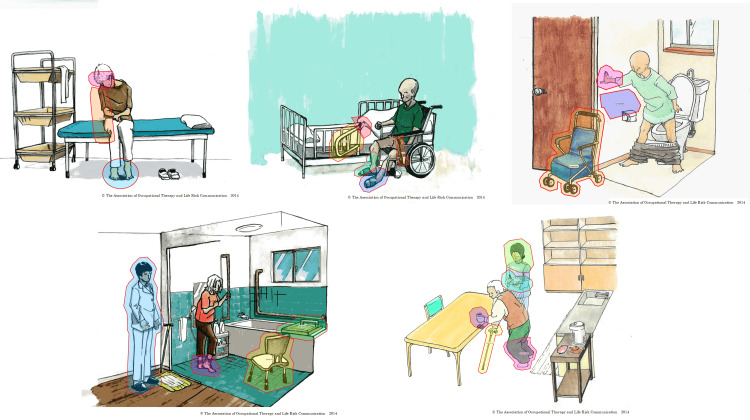
Area of interest (AOI) in five scenes of TP-KYT Each colored and outlined area represents a specific AOI corresponding to a scorable physical hazard that was analyzed in this study. TP-KYT: Time Pressure-Kiken Yochi Training Image Source: The Association of Occupational Therapy and Life Risk Communication, the original copyright holder; used as per the society's guidelines (http://seikatsurisk.kenkyuukai.jp/information/information_detail.asp?id=107582)

An area of interest (AOI) was established on each image to capture the eye movement status of the hazard scenes in the TP-KYT images. Our analysis focused on the potential discrepancy between visually fixating on a hazard and cognitively perceiving it as a risk. To ensure objective measurement of gaze, AOIs were strictly defined around scorable physical objects or specific parts of the environment depicted in the scenes (Figure 1). Consequently, conceptual hazards identified in the TP-KYT scoring system (e.g., those related to a patient's condition or the absence of people around), which did not have a discrete physical location in the image, were excluded from the said eye-tracking analysis. This was necessary since it is impossible to map gaze data to a non-spatial concept, and our objective was to analyze attention directed toward specific visual cues. The AOIs were defined as follows: ‘take note’ referred to the area encompassing the therapist's hands and the clipboard they were writing on; ‘therapist’ referred to the full body of the therapist figure; ‘hand’ referred to the patient's hand not holding the walker; ‘heel’ referred to the patient's heels being lifted off the floor. The boundaries for each AOI were manually drawn using the polygon tool in the Tobii Pro Lab software version 1.171.34906 (Tobii AB). Each polygon was carefully delineated to encompass the entire visible area of the corresponding physical hazard (e.g., the wheelchair brake, the bed fence) as depicted in the TP-KYT illustration.

In this study, we focused on situations in which participants looked at a hazardous situation with gaze fixation but did not perceive it as dangerous, and a more apparent distinction between gaze fixation and saccades was required. The Tobii I (Attention) filter is suitable for distinguishing between gaze fixation and saccades. Therefore, we followed the Tobii I-VT (Attention) filter's default settings in the Tobii Pro Lab gaze analysis software (version 1.171.34906). 

Setting up the gaze analysis environment

**Figure 2:**
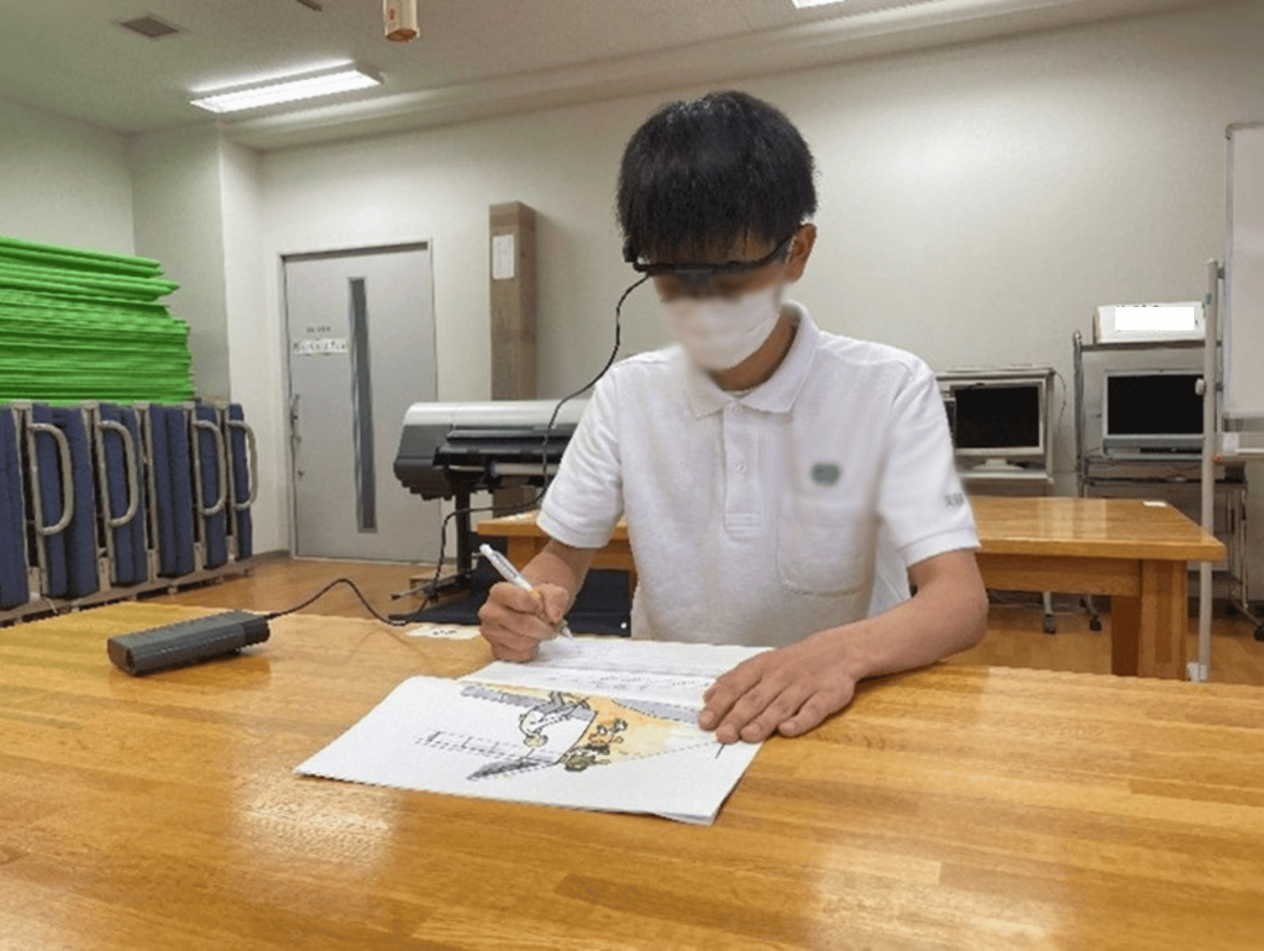
The environmental setting for implementation

We used a standard desk and chair for the measurements, and the room was brightly lit, so the illustrations could be easily seen (Figure 2). When the gaze information was collected with the subject wearing the eye camera, we ensured that the entire figure was recorded.

Data collection procedures

After obtaining consent to participate in the study, measurements were taken individually. The participants completed the calibration while wearing the eye tracker. They then completed the TP-KYT by checking for hazards in five situations and describing them according to the implementation procedures.

Data analysis

Comparison of Hazard Perception

To determine whether there were differences in risk perception between the groups, we compared the TP-KYT scores. Specifically, we analyzed both the total score (the sum of scores from all five scenes) and the individual scores for each of the five scenes between healthcare professionals and students. We also compared whether there were differences in the total scores between men and women.

Video Image Analysis With an Eye Tracker

We analyzed the video recorded by the eye tracker using an end time of 10 seconds, which is the detection time of a hazardous situation in a TP-KYT scene, as the standard, and the time of interest (TOI) as the 10-second period leading up to the end time. To determine whether there was a difference in the fixation time for each AOI in each scene, we extracted the total duration of fixation in the AOI from Tobii Pro Lab eye-tracker gaze analysis software and compared the gaze dwell time. Next, we compared the total amplitude of saccades within 10 seconds to compare the total eye movement distance in each TP-KYT scene. The results were further classified into four categories based on gaze fixation and risk perception at each AOI: fixation and cognition (FC), fixation and non-cognition (FnC), non-fixation and cognition (NFC), and non-fixation and non-cognition (MFC). Differences in the AOI between healthcare professionals and students were examined for FnC.

For our analysis, risk perception was operationally defined as a participant correctly identifying a hazard within an AOI on the TP-KYT answer sheet, thereby receiving a score for that hazard. Conversely, ‘non-cognition’ was defined as the failure to identify and mark the scored hazard. Additionally, the NFC category represented instances where a participant correctly scored a hazard on the TP-KYT sheet, but the gaze duration on the corresponding AOI was too brief to be classified as a fixation by our predefined threshold. This suggests that the hazard was identified through a rapid visual intake and cognitive judgment, rather than sustained foveal attention.

Statistical analysis

The Mann-Whitney U test was used for TP-KYT scores between healthcare professionals and students, between male and female participants for each situation, and for total scores. In addition, because there was a difference in the proportions of male and female participants between the two groups in this study, we compared whether there was a difference in the total scores between them.

Each AOI's fixation time and total saccade amplitude were subjected to the Mann-Whitney U test for each scene. Next, to examine whether FnC differed by experience, we checked the risk ratios of unnoticed situations between healthcare professionals and students regarding whether they were aware of the danger points during gaze cessation. While all four categories were classified, our primary statistical analysis focused on the risk ratios for FnC, as this category most directly addresses our research question regarding the failure to perceive a risk despite fixating on it.

IBM SPSS Statistics for Windows, version 27.0 (IBM Corp., Armonk, New York, United States) was used for all statistical analyses, and all analyses were considered significant at a risk rate of less than 5%.

## Results

**Figure 3:**
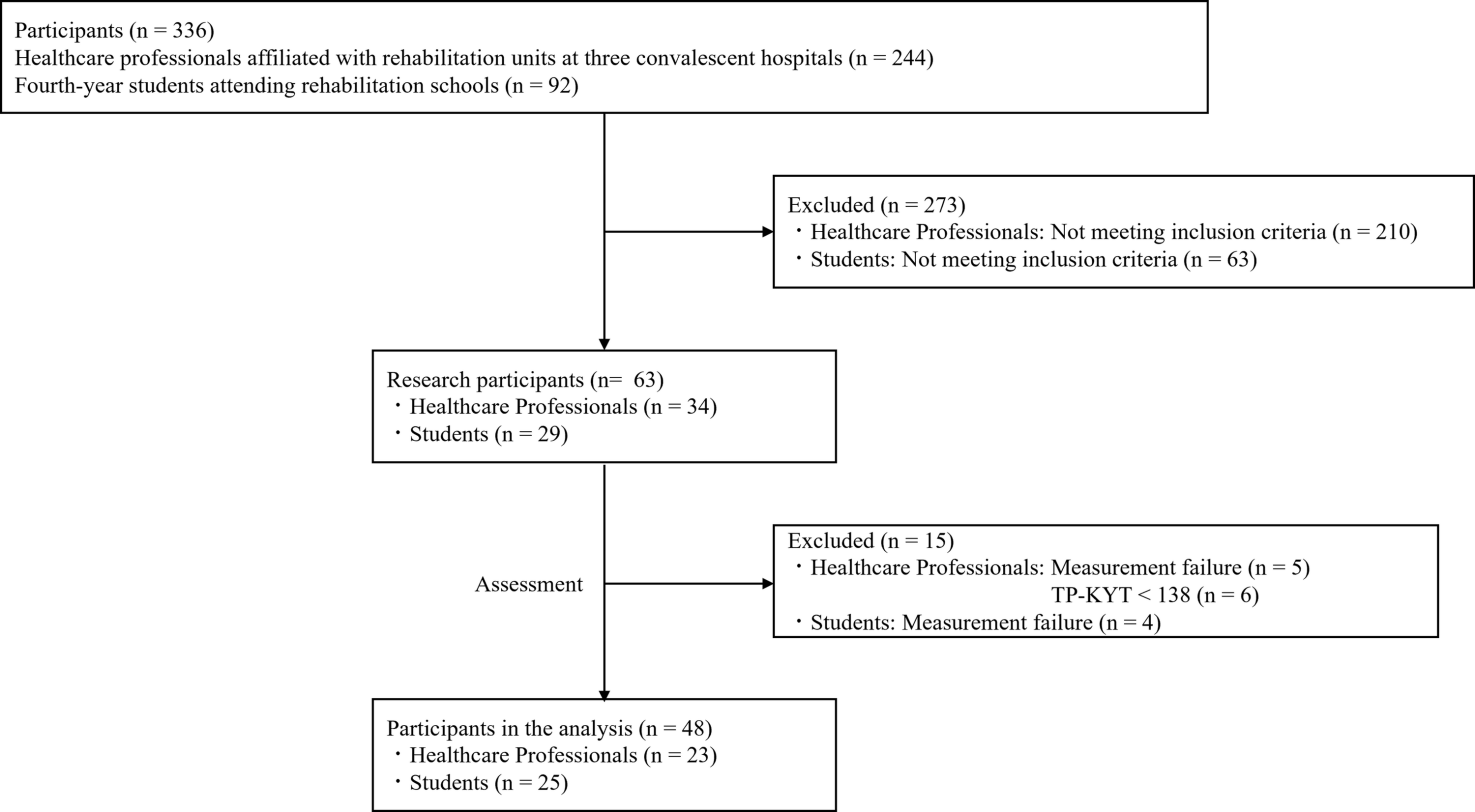
Flow diagram of participants TP-KYT, Time Pressure - Kiken Yochi Training System

Of the subjects, 23 were healthcare professionals with work experience, and 25 were students (Figure 3). Table 1 presents the essential characteristics of the study participants.

**Table 1:** Participants’ characteristics

Parameters	Healthcare Professionals (n = 23)	Students (n = 25)
Age (years), mean ± SD	32.83 ± 5.45	21.68 ± 1.15
Duration of experience (month), mean ± SD	118.04 ± 66.34	-
Gender, n (%)		
Men	16 (69.57)	9 (36.00)
Women	7 (30.43)	16 (64.00)
Job role, n (%)		
Occupational Therapist	11 (47.83)	-
Physical Therapist	10 (43.48)	-
Nurse	2 (8.69)	-

Comparison of TP-KYT scores between the two groups by sex

Healthcare professionals scored higher than students in all scenarios and total scores (Tables 2, 3). The total score of the TP-KYT did not differ between male and female participants (172.60 ± 64.91 points in male vs. 148.48 ± 65.04 points in female; p = .21).

**Table 2:** Comparison of total score in TP-KYT *Mann-Whitney U test.  **p < 0.01. TP-KYT, Time Pressure - Kiken Yochi Training System

	Healthcare Professionals (n = 23) , mean ± SD	Students (n = 25), mean ± SD	Test Statistic*	p-Value
Total score	208.70 ± 44.65	117.20 ± 48.74	U = 46.00	< .01**

**Table 3:** Comparison of scores for each scene in TP-KYT ^#^Mann-Whitney U test. *p < 0.05 **p < 0.01. TP-KYT, Time Pressure - Kiken Yochi Training System

Scene	Score of Healthcare Professionals (n = 23), mean ± SD	Score of Students (n = 25), mean ± SD	Test Statistic^#^	p-Value
Scene 1: sitting	30.00 ± 14.38	19.80 ± 14.75	U = 183.00	0.03*
Scene 2: patient transfer from the bed	49.78 ± 22.33	18.82 ± 16.41	U = 92.50	< .01**
Scene 3: patient transfer to the toilet	53.26 ± 15.78	26.40 ± 17.23	U = 81.50	< .01**
Scene 4: patient transfer to the bathroom	35.65 ± 22.22	22.00 ± 20.67	U = 179.00	0.02*
Scene 5: patient transfer from the kitchen	40.00 ± 18.34	23.60 ± 17.23	U = 152.50	< .01**

Comparison of total duration of fixation and amplitude of saccades per scene for each AOI

The total fixation duration in the AOI was shorter for the therapists in Scenario 4 (p = .02). However, the fixation duration for the other AOIs in each scene was the same (Table 4). The total amplitude angle of the saccade in each scene did not differ between healthcare professionals and students (Table 5).

**Table 4:** Comparison of total duration of fixation across AOIs for each scene **Mann-Whitney U test. *p < 0.05 AOI, area of interest

Scene	AOI	Total duration of fixation in AOI (milliseconds)		Test Statistic**	p-Value
		Healthcare professionals (n = 23)	Students (n = 25)		
Scene 1: sitting	Body tilt	0.85 ± 1.06	1.18 ± 1.02	U = 354.50	0.16
	Face down	0.39 ± 0.49	0.40 ± 0.86	U = 271.50	0.72
	Sole	0.71 ± 0.77	1.10 ± 0.83	U = 381.50	0.05
Scene 2: patient transfer from the bed	Brake	0.41 ± 0.66	0.28 ± 0.56	U = 260.50	0.53
	Fence fix	0.91 ± 1.00	0.53 ± 0.65	U = 231.00	0.23
	Fence open	0.37 ± 0.56	0.55 ± 0.75	U = 319.50	0.48
	Left sole	0.54 ± 0.67	0.79 ± 0.81	U = 328.00	0.39
	Leg position	0.19 ± 0.51	0.12 ± 0.33	U = 288.00	0.99
Scene 3: patient transfer to the toilet	Hand	0.48 ± 0.76	0.63 ± 0.75	U = 321.50	0.46
	No handrail	0.56 ± 0.53	0.58 ± 0.93	U = 245.50	0.38
	Walker	1.03 ± 0.62	1.07 ± 1.02	U = 259.00	0.56
Scene 4: patient transfer to the bathroom	Bathboard	0.50 ± 0.47	0.41 ± 0.46	U = 251.50	0.45
	Showerchair	0.95 ± 0.50	0.71 ± 0.49	U = 212.00	0.12
	Socks	0.63 ± 0.80	0.65 ± 0.77	U = 303.00	0.73
	Therapist	0.73 ± 0.69	0.36 ± 0.50	U = 174.50	0.02*
Scene 5: patient transfer from the kitchen	Both hands	0.30 ± 0.37	0.74 ± 0.92	U = 349.50	0.19
	Standing position	0.56 ± 0.81	0.61 ± 0.53	U = 341.50	0.26
	Take note	0.64 ± 0.61	0.49 ± 0.59	U = 244.00	0.36
	Therapist	0.42 ± 0.82	0.29 ± 0.43	U = 298.00	0.81
	Weight on cane	0.84 ± 1.21	0.92 ± 1.19	U = 303.50	0.73

**Table 5:** Comparison of total amplitude of saccades for each scene *Mann-Whitney U test

Scene	Total amplitude of saccades (degree)		Test Statistic*	p-Value
	Healthcare professionals (n = 23), mean ± SD	Students (n = 25), mean ± SD		
Scene 1: sitting	98.20 ± 36.35	96.20 ± 33.30	U = 277.00	0.83
Scene 2: patient transfer from the bed	56.77 ± 24.26	51.29 ± 28.66	U = 245.00	0.38
Scene 3: patient transfer to the toilet	85.82 ± 34.89	79.72 ± 24.29	U = 256.00	0.52
Scene 4: patient transfer to the bathroom	94.40 ± 34.65	82.30 ± 26.70	U = 244.00	0.37
Scene 5: patient transfer from the kitchen	59.90 ± 32.75	60.24 ± 33.22	U = 285.00	0.96

 Gaze fixation and risk perception

Risk perception differed between healthcare professionals and students across several AOIs (Table 6). An analysis of fixation without risk perception (FnC) revealed that students were generally more likely to exhibit FnC than healthcare professionals. The highest overall risk ratio for FnC at the scene level was observed in Scene 3 (risk ratio (RR): 2.68, 95%CI: 1.46-4.91), followed by Scene 2 (RR: 2.09, 95%CI: 1.41-3.11).

**Table 6:** Comparison of differences in risk perception for each AOI **Fisher's exact test statistics for the Fisher-Freeman-Halton Exact Probability Tests; *p < 0.05. AOI, area of Interest

Scene	AOI	Job role	Fixation and cognition (FC)		Fixation and non-cognition (FnC)		Non-fixation and cognition (NFC)		Non-fixation and non-cognition (MFC)		Test statistic**	p-Value
			Frequency	Percentage	Frequency	Percentage	Frequency	Percentage	Frequency	Percentage		
Scene 1: sitting	Body tilt	Healthcare professionals	11	47.83	4	17.39	1	4.34	7	30.43	7.83	0.05
		Students	10	40.00	12	48.00	1	4.00	2	8.00		
	Face down	Healthcare professionals	3	13.04	8	34.78	3	13.04	9	39.13	2.63	0.45
		Students	1	4.00	12	48.00	1	4.00	11	44.00		
	Sole	Healthcare professionals	12	52.17	6	26.09	5	21.74	0	0.00	8.02	0.05*
		Students	13	52.00	11	44.00	0	0.00	1	4.00		
Scene 2: patient transfer from the bed	Brake	Healthcare professionals	6	26.09	4	17.39	3	13.04	10	43.48	9.42	0.02*
		Students	0	0.00	9	36.00	2	8.00	14	56.00		
	Fence fix	Healthcare professionals	10	43.48	6	26.09	1	4.34	6	26.09	9.36	0.03*
		Students	2	8.00	12	48.00	1	4.00	10	40.00		
	Fence open	Healthcare professionals	6	26.09	4	17.39	3	13.04	10	43.48	11.15	0.01*
		Students	1	4.00	12	48.00	0	0.00	12	48.00		
	Left sole	Healthcare professionals	13	56.52	1	4.34	7	30.43	2	8.70	2.41	0.49
		Students	12	48.00	4	16.00	5	20.00	4	16.00		
	Leg position	Healthcare professionals	4	17.39	0	0.00	0	0.00	19	82.61	0.00	1.00
		Students	5	20.00	0	0.00	1	4.00	19	76.00		
Scene 3: patient transfer to the toilet	Hand	Healthcare professionals	10	43.48	1	4.34	2	8.70	10	43.48	5.82	0.12
		Students	8	32.00	6	24.00	0	0.00	11	44.00		
	No handrail	Healthcare professionals	15	65.22	2	8.70	1	4.34	5	21.74	10.73	0.01*
		Students	6	24.00	10	40.00	2	8.00	7	28.00		
	Walker	Healthcare professionals	14	60.87	7	30.43	2	8.70	0	0.00	7.12	0.07
		Students	10	40.00	13	52.00	0	0.00	2	8.00		
Scene 4: patient transfer to the bathroom	Bathboard	Healthcare professionals	5	21.74	10	43.48	1	4.34	7	30.43	1.74	0.63
		Students	3	12.00	13	52.00	0	0.00	9	36.00		
	Showerchair	Healthcare professionals	6	26.09	17	73.91	0	0.00	0	0.00	9.34	0.02*
		Students	1	4.00	21	84.00	0	0.00	3	12.00		
	Socks	Healthcare professionals	9	39.13	2	8.70	6	26.09	6	26.09	0.60	0.90
		Students	11	44.00	2	8.00	4	16.00	8	32.00		
	Therapist	Healthcare professionals	10	43.48	10	43.48	1	4.34	2	8.70	8.25	0.04*
		Students	4	16.00	13	52.00	0	0.00	8	32.00		
Scene 5: patient transfer from the kitchen	Both hands	Healthcare professionals	1	4.34	13	56.52	0	0.00	9	39.13	0.00	1.00
		Students	1	4.00	14	56.00	1	4.00	9	36.00		
	Standing position	Healthcare professionals	5	21.74	10	43.48	1	4.34	7	30.43	1.85	0.61
		Students	8	32.00	12	48.00	0	0.00	5	20.00		
	Take note	Healthcare professionals	11	47.83	4	17.39	3	13.04	5	21.74	9.22	0.03*
		Students	4	16.00	12	48.00	1	4.00	8	32.00		
	Therapist	Healthcare professionals	2	8.70	6	26.09	0	0.00	15	65.22	1.13	0.57
		Students	1	4.00	10	40.00	0	0.00	14	56.00		
	Weight on cane	Healthcare professionals	9	39.13	4	17.39	7	30.43	3	13.04	1.38	0.71
		Students	12	48.00	5	20.00	4	16.00	4	16.00		

A detailed breakdown of the FnC percentages and RRs for each specific AOI is provided in Tables 7, 8. The AOI with the highest FnC risk for students compared to healthcare professionals was ‘no handrail’ in Scene 3 (RR: 5.31, 95%CI: 1.37-20.62). The next highest was ‘take note’ in Scene 5 (RR: 2.81, 95%CI: 1.16-6.82). Scene 2 contained three AOIs where students had a significantly higher risk of FnC: ‘brake’ (RR: 2.50, 95%CI: 1.17-5.34), ‘fence fixed’ (RR: 2.29, 95%CI: 1.17-4.46), and ‘fence open’ (RR: 2.31, 95%CI: 1.06-5.01). Finally, the ‘lower chair’ in Scene 4 also exhibited a higher FnC risk for students (RR: 1.29, 95%CI: 1.00-1.67).

**Table 7:** Percentage of gaze fixation and no risk perception across AOIs for each scene a = number of AOIs×n, x = number of occurrences AOI, area of interest

		Healthcare professionals (n = 23)			Students (n = 25)		
Scene	AOI	Total (a)	With gaze fixation, x (％)	Fixation and non-cognition (FnC), x (%)	Total (a)	With gaze fixation, x (％)	Fixation and non-cognition (FnC), x (%)
Scene 1: sitting	Body tilt	69	15 (21.74)	4 (26.67)	75	22 (29.33)	12 (54.55)
	Face down	69	11 (15.94)	8 (72.73)	75	13 (17.33)	12 (92.31)
	Sole	69	18 (26.09)	6 (33.33)	75	24 (32.00)	11 (14.67)
Scene 2: patient transfer from the bed	Brake	115	10 (8.70)	4 (40.00)	125	9 (7.20)	9 (100.00)
	Fence fix	115	16 (13.91)	6 (37.50)	125	14 (11.20)	12 (85.71)
	Fence open	115	10 (8.70)	4 (40.00)	125	13 (10.40)	12 (92.31)
	Left sole	115	14 (12.17)	1 (7.14)	125	16 (12.80)	4 (25.00)
	Leg position	115	4 (3.48)	4 (100.00)	125	5 (4.00)	5 (100.00)
Scene 3: patient transfer to the toilet	Hand	69	11 (15.94)	1 (9.09)	75	14 (18.67)	6 (42.86)
	No handrail	69	17 (24.64)	2 (11.76)	75	16 (21.33)	10 (62.50)
	Walker	69	21 (30.43)	7 (33.33)	75	23 (30.67)	13 (56.52)
Scene 4: patient transfer to the bathroom	Bathboard	92	15 (16.30)	10 (66.67)	100	16 (16.00)	13 (81.25)
	Showerchair	92	23 (25.00)	17 (73.91)	100	22 (22.00)	21 (95.45)
	Socks	92	11 (11.96)	2 (18.18)	100	13 (13.00)	2 (15.38)
	Therapist	92	20 (21.74)	10 (50.00)	100	17 (17.00)	13 (76.47)
Scene 5: patient transfer from the kitchen	Both hands	115	14 (12.17)	13 (92.86)	125	15 (12.00)	14 (93.33)
	Standing position	115	15 (13.04)	10 (66.67)	125	20 (16.00)	12 (60.00)
	Take note	115	15 (13.04)	4 (26.67)	125	16 (12.80)	12 (75.00)
	Therapist	115	8 (6.96)	6 (75.00)	125	11 (8.80)	10 (90.91)
	Weight on cane	115	13 (11.30)	4 (30.77)	125	17 (13.60)	5 (29.41)

**Table 8:** Risk ratio for no risk perception during gaze fixation across AOIs for each scene *Chi-squared tests. **p < 0.01. Risk ratio could not be calculated for the ‘Leg position’ AOI as there were zero events in the reference group AOI, area of interest

Scene	AOI	For no risk perception during gaze fixation		Test Statistic*	p-Value
		Risk ratio for students/healthcare professionals	95% confidence interval for risk ratios		
Scene 1: sitting	Body tilt	2.05	0.81 - 5.14	χ² (1) = 2.82	0.09
	Face down	1.27	0.86 - 1.88	χ² (1) = 1.65	0.20
	Sole	1.38	0.63 - 3.01	χ² (1) = 0.67	0.41
Scene 2: patient transfer from the bed	Brake	2.50	1.17 - 5.34	χ² (1) = 7.89	< .01**
	Fence fix	2.29	1.17 - 4.46	χ² (1) = 7.23	< .01**
	Fence open	2.31	1.06 - 5.01	χ² (1) = 7.30	< .01**
	Left sole	3.50	0.44 - 27.75	χ² (1) = 1.71	0.19
	Leg position	-	-	-	-
Scene 3: patient transfer to the toilet	Hand	4.71	0.66 - 33.61	χ² (1) = 3.48	0.06
	No handrail	5.31	1.37 - 20.62	χ² (1) = 9.17	< .01**
	Walker	1.70	0.84 - 3.43	χ² (1) = 2.38	0.12
Scene 4: patient transfer to the bathroom	Bathboard	1.22	0.78 - 1.87	χ² (1) = 0.86	0.35
	Showerchair	1.29	1.00 - 1.67	χ² (1) = 3.97	0.05
	Socks	0.85	0.14 - 5.06	χ² (1) = 0.03	0.86
	Therapist	1.53	0.92 - 2.55	χ² (1) = 2.73	0.10
Scene 5: patient transfer from the kitchen	Both hands	1.01	0.82 - 1.23	χ² (1) < 0.01	0.96
	Standing position	0.90	0.54 - 1.49	χ² (1) = 0.16	0.69
	Take note	2.81	1.16 - 6.82	χ² (1) = 7.24	< .01**
	Therapist	1.21	0.78 - 1.89	χ² (1) = 0.88	0.35
	Weight on cane	0.96	0.32 - 2.87	χ² (1) < 0.01	0.94

## Discussion

In this study, we investigated (i) whether the fixation duration on risk depends on experience and (ii) whether the risk of missing a fall hazard situation is reduced by experience. Regarding the first point, we found that the fixation duration and saccade amplitude did not differ by experience when recognizing risks under time pressure. Regarding the second point, students' performance differed according to their experience, and they were more likely to miss the risk of falling, even though they were looking at a risky situation. These data provide information for understanding students' risk perceptions and may provide a rationale for more objective educational interventions.

Selection of participants

The characteristics of the participants were examined, revealing no difference in the total TP-KYT score between male and female participants and no effect of subject demographics on the score. However, when the scores of each scene and the total score were compared between the healthcare professionals and the students, differences were found in the total score and all scores of each scene. The cutoff values for the total score of the TP-KYT were 213 points for experts and 138 points for competitors. The mean total score of healthcare professionals in this study was 208.7 points, close to the expert's cutoff. The mean total score of the fourth-year students was 117.2 points, lower than that of experts. Therefore, healthcare professionals and fourth-year students are appropriate participants for this study.

Difference between duration of fixation and amplitude of saccades with experience

Overall, the fixation duration and amplitude of saccades for risk did not significantly differ by experience across most AOIs. However, a notable exception was observed for the "therapist" AOI in Scene 4, where students exhibited a significantly shorter fixation duration. A previous study reported that the duration of fixation and amplitude of saccades for expert and non-expert patients showed longer fixation durations and greater amplitudes for non-expert patients [15,20,21]. However, another report found no difference in the fixation duration between expert and nonexpert patients [14]. In the present study, although a difference was observed in the therapist, a subtotal of the AOI of Scene 4, non-expert students showed shorter fixation durations. There were no differences in fixation duration among the other AOI subtasks. Students with less clinical experience tend to have longer fixation durations because they are more careful in gathering information about important parts [14,17]. The experts tended to have longer fixation durations for the most exciting parts and tended to view the entire area with peripheral vision [22]. In this study, the AOI was set for a dangerous area that was also a dangerous area of high interest to the expert; therefore, it is likely that the expert judged the danger using peripheral vision around the area where the AOI was set. These results suggest that in the hazard detection task of TP-KYT, the students' behavior of carefully searching for information and the experts’ behavior of searching for a hazard area of high interest were captured as similar eye movements. This may explain why no difference was observed in the fixation duration or saccades' amplitude.

As for the difference in the AOI item "therapist" in Scene 4, it can be inferred that the students did not fully consider the therapist's proximity as a safety factor, such as the ability to intervene quickly in case of a fall. The presence of a therapist nearby may have led them to feel a false sense of security without assessing the spatial relationship as a risk. The difference in judgments may result from differences in risk judgments between the two groups since experts can make risk judgments based on environmental and situational cues when they perceive danger [23]. Scene 5 also has an AOI item for the therapist. Still, this item has both the risks of the distance of the person who can respond and the fact that they are looking at the note, and it can be inferred that the students quickly perceived the risks of the therapist AOI item "therapist." This point is discussed in the following FnC discussion.

Difference in FnC by experience

The results for FnC are interesting. In all scenes, the FnC was higher for students than for experts, especially in Scenes 2 and 3, where the RR was more than twice as high. In Scenarios 2 and 3, the RR was more than twice as high. In each AOI of Scene 2, students showed higher FnC in the three AOIs: "brake,” "fence fixed,” and "fence open.” In addition, "no handrail" showed the highest FnC of all AOIs in each AOI of Scene 3. These are often risks caused by the relationship between the subject and the environment. The results suggest that students tend to be less aware of the dangers of safety aids, such as brakes, fences, and handrails. In a previous study comparing the responses of nursing students and nurses to hazardous situations, it was reported that students did not reach the point of thinking to focus on the environment, considering the subject's condition and situation [14]; it is possible that students believed that the mere presence of assistive devices made them safe and could not identify them as a risk. In a previous study that determined the risk prediction ability included in the TP-KYT, it was shown that the ability to predict the environment, patient ability, and human environment, in that order, can be cultivated through experience [24], which supports the results of this study.

Although there was no difference in the FnC of "shower chair" in Scene 4 and "take note" in Scene 5, there was a difference in the FnC between students and experts regarding AOI. These two AOIs are the physical and human environments and are risks caused by the positional relationship and distance to the object. In the human environment, as with the three AOIs of "break,” "fence fix,” and "fence open" in Scenes 2 and 3, it is believed that the students did not reach the point of thinking to focus on the environment, considering the subject's condition and situation. The therapist's presence may have caused the participant to judge that they were not at risk, and the distance behind this judgment may not have been used for risk perception.

This study investigated students' risk perceptions using an eye tracker. Our findings suggest that students were more likely to experience FnC than experts. The students appeared to be characterized by an inability to make immediate judgments about the relationship between environmental factors, including the human environment, and the object [14], and could not view the relationship between the object and ecological factors as contextual. In risk education for students, recognizing risk with contextual information is essential for those with little teaching experience.

Limitations

This study has several limitations. First, the small number of participating sites and the recruitment from a single training school for students may have introduced selection bias. Second, the stress of performing the TP-KYT under time constraints while wearing equipment may have influenced the results. Third, although the student participants were in their final year of training, it is uncertain whether these results can be generalized to newcomers in the medical field. Fourth, applying a TP-KYT competence score-based exclusion criterion (≥138) only to the healthcare professional group introduces a potential selection bias, possibly making this group more uniformly competent and affecting comparisons. This was a deliberate design choice to contrast learners with professionals deemed competent, but it limits the generalizability of our findings. Future studies might consider alternative methods for defining expertise. Fifth, for some AOIs, the low number of observed FnC events resulted in wide 95% CIs for some RRs, indicating substantial statistical uncertainty. Therefore, these specific findings should be interpreted with caution. Sixth, as this study was exploratory in nature, multiple comparisons were made across numerous AOIs without a formal statistical correction like the Bonferroni method. Thus, the findings for individual AOIs should be viewed as preliminary and require confirmation in future hypothesis-driven research. Finally, as the TP-KYT was developed in Japan, the findings must be considered within the context of the general rehabilitation situation in Japanese healthcare facilities.

## Conclusions

This study suggests that, while students' visual search patterns may be similar to those of experienced healthcare professionals, the former are significantly more likely to fail to perceive a risk despite fixating on it. This discrepancy was most pronounced for hazards requiring contextual understanding of the relationship between the patient, the environment, and safety equipment. These findings underscore the need for educational interventions that move beyond simple hazard identification to explicitly train students in contextual risk assessment to improve patient safety.
